# Concordance of folate receptor-α expression between biopsy, primary tumor and metastasis in breast cancer and lung cancer patients

**DOI:** 10.18632/oncotarget.7856

**Published:** 2016-03-02

**Authors:** Leonora S. F. Boogerd, Martin C. Boonstra, Ann-Jean Beck, Ayoub Charehbili, Charlotte E.S. Hoogstins, Hendrica A. J. M. Prevoo, Sunil Singhal, Philip S. Low, Cornelis J. H. van de Velde, Alexander L. Vahrmeijer

**Affiliations:** ^1^ Department of Surgery, Leiden Univeristy Medical Center, Leiden, The Netherlands; ^2^ Department of Thoracic Surgery, Hospital of The University of Pennsylvania, Philadelphia, PA, USA; ^3^ Department of Chemistry and Center for Drug Discovery, Purdue University, West Lafayette, IN, USA

**Keywords:** diagnosis, personalized medicine, targeting, oncology, biomarker, Pathology Section

## Abstract

Folate receptor alpha (FRα) is known to be upregulated in a variety of cancers, including non-small cell lung cancer (NSCLC) and breast cancer. To ensure reliable implementation of diagnostic- and therapeutic agents, concordance of FRα expression between biopsy, primary tumor and metastases is important. Using immunohistochemistry (Mab 26B3.F2) these concordances were investigated in 60 NSCLC and 40 breast cancer patients. False positivity of FRα expression on breast and lung cancer biopsies was limited to less than 5%. In NSCLC, FRα expression was shown in 21/34 adenocarcinomas and 4/26 squamous cell carcinomas (SCC). Concordance of FRα expression between biopsy and primary tumor was achieved in respectively 83% and 91% of adenocarcinomas and SCCs. Approximately 80% of all local and distant metastases of NSCLC patients showed concordant FRα expression as their corresponding primary tumor. In breast cancer, FRα positivity was shown in 12/40 biopsies, 20/40 lumpectomies and 6/20 LN metastases, with concordance of 68% between biopsy and primary tumor and 60% between primary tumor and LN metastases. In conclusion, this study shows high concordance rates of FRα expression between biopsies and metastases compared to primary NSCLC and breast cancers, underscoring the applicability of FRα-targeted agents in these patients.

## INTRODUCTION

The introduction of molecular targeted oncologic therapies has created the need to select patients with the right expression patterns to achieve optimal results [1]. Currently, the molecular markup of tumors can be determined using (real-time or quantitative reverse transcription) polymerase chain reaction, microRNA detection, or immunohistochemistry(IHC). Immunohistochemical staining of tumor tissue is the preferred method, since it provides insight about the expression pattern of specific membrane-bound proteins and, in addition, is a relatively easy and inexpensive technique. Immunohistochemical staining of tumor biopsies is already implemented in clinical practice and used as an efficient selection method in cancer care in, for example, breast cancer patients [2]. Concordance of biomarker expression between biopsy, primary tumor and (distant) metastasis is important for appropriate application of tumor-targeted modalities; e.g. Positron Emission Tomography (PET) or Single Photon Emission Computed Tomography (SPECT)-targeted agents or targeted therapies. For example, a biopsy can turn out to be positive for a certain biomarker while the distant metastasis showed to be negative in most of the cases, resulting in suboptimal applications/results of the targeted approaches. In this study, we investigate these concordances in NSCLC and breast cancer patients and generate insight for the reliable application of FRα-targeted agents in these patients.

In the past decades, several membrane-located proteins were discovered that could successfully be targeted, i.e. estrogen/progesterone receptor (ER/PR) in breast cancer patients and epidermal growth factor receptor (EGFR) in lung cancer patients [2, 3]. Another potential target that holds great promise is folate receptor alpha (FRα). This glycosylphosphatidylinositol (GPI)-anchored cell membrane protein was first described on a human ovarian cancer cell line in 1991 and is part of the folate receptor (FR) family [4, 5]. Folic acid and 5-methyltetrahydrofolate, the major circulating form of folate, bind with high affinity to these FRs [6]. Folate, the water soluble vitamin B9, is involved in one-carbon metabolic reactions and is therefore required in the biosynthesis of the nucleotide bases thymine and uracil. The essential role of folate in growth, proliferation and survival of cells reflects the importance of folate during growth and sustenance of tumor cells [7, 8]. In total, four isoforms of FR are described of which FRβ and FRγ are sited on hematopoietic cells and overexpressed on cancer cells of hematopoietic origin [7]. The isoform FRβ is also found on activated macrophages, while the function of FRδ remains unclear. FRα is normally located on the apical surface of a restricted number of healthy epithelial tissues, such as proximal kidney tubules, fallopian tube and type I and II pneumocytes of the lung [9]. Research on FRα has focused on identification of tumor types that show FRα overexpression and a recent study showed that 40% of epithelial tumors show FRα overexpression, including (non-mucinous) ovarian, kidney, uterine, colon, lung and breast cancer [10].

Lung cancer is the number one cause of cancer death worldwide with a poor five-year survival estimated at 15%. This disease can be divided in NSCLC, contributing to 85% of all lung cancers, and small cell lung cancer (SCLC). NSCLC is further subdivided by histological subtype, of which adenocarcinoma (40%), squamous cell carcinoma (SCC) (25-30%) and large-cell carcinoma (10-15%) are the most prevalent [11]. Tumor-targeted therapies have already been extensively studied in lung cancer patients, which resulted in implementation of such therapies, e.g. bevacizumab, erlotinib and gefitinib, in the treatment guideline of lung cancer disease [12]. Overexpression of FRα in lung cancer is mainly restricted to NSCLC and can therefore be an interesting target for many lung cancer patients [13, 14]. In breast cancer, treatment is already personalized by molecular protein expression of the tumor [2]. The outcome of breast cancer patients is significantly improved by targeted treatment focused on hormone receptor (HR) positive, or human epidermal growth factor receptor 2 (HER2) positive tumors. However, 15% of all breast cancers show no expression of either ER/PR or HER2 and are considered triple negative (TN) breast cancers. This type of breast cancer has the worst prognosis amongst all breast cancers [15]. Previous studies reported FRα overexpression on especially the majority of TN breast cancers and therefore, FRα could be an interesting target for this specific group of patients [16].

The aim of the present study is to determine FRα expression on both NSCLC and breast cancers and to determine concordance between FRα expression on biopsy, primary tumor and corresponding local and distant metastatic tissue.

## RESULTS

### Patient- and tumor characteristics

Patient- and histopathological characteristics of all NSCLC and breast cancer patients are summarized in Table 1. In short, mean age of breast and NSCLC patients was respectively 56 and 63.5 years at diagnosis. The majority of breast cancer patients displayed pT1 or pT2 stage tumors and all of them underwent a lumpectomy. The majority of NSCLC patients displayed pT4 tumors and IASCL stage IV. Of both NSCLC and breast cancer patients approximately 70% showed lymph node involvement, i.e. nodal stage N1-2. The cohort of breast cancer patients consisted of 15 TN breast cancers with a majority of high grade tumors, e.g. 21 out of 40 tumors showed differentiation grade III.

**Table 1:** Patient and tumor characteristics

Variable	NSCLC, adenocarcinoma No. (%)	NSCLC, SCC No. (%)	Breast cancer No. (%)
**All patients**	34 (57)	26 (43)	40
**Mean age at diagnosis (years)**	64.5	62.5	56
**Sex**			
male	21 (87.5)	20 (77)	0 (0)
female	13 (12.5)	6 (23)	40 (100)
**Tumor size**			
pT1	10 (29)	3 (12)	23 (57.5)
pT2	5 (15)	6 (23)	16 (40)
pT3	4 (12)	4 (15)	1 (2.5)
pT4	15 (44)	13 (50)	0 (0)
**Nodal Stage**			
Nx	0 (0)	0 (0)	1 (2.5)
N0	7 (21)	5 (19)	12 (30)
N1-2	27 (79)	21 (81)	27 (67.5)
**IASCL stage**			
IA	4 (12)	1 (4)	
IB	2 (6)	2 (8)	
IIA	3 (9)	4 (15)	
IIB	2 (6)	0 (0)	
IIIA	7 (20)	10 (38)	
IIIB	1 (3)	1 (4)	
IV	15 (44)	8 (31)	
**Marker status**			
HR status			
positive			19 (47.5)
negative			21 (52.5)
HER2 status			
positive			17 (42.5)
negative			23 (57.5)
TN			
yes			15 (37.5)
no			25 (62.5)
**Histology**			
Ductal			36 (90)
Lobular			4 (10)
**Tumor grade**			
NE			4 (10)
Grade 1			2 (5)
Grade 2			13 (32.5)
Grade 3			21 (52.5)
**Neo-adjuvant therapy**			
Yes	14 (41)	8 (31)	6 (15)
No	20 (59)	18 (69)	34 (85)

Abbreviations: NSCLC non-small cell lung cancer; SCC squamous cell carcinoma; pT pathological tumor stage, N node status, IASCL International Association for the Study of Lung Cancer; HR hormone receptor, including progesterone and estrogen receptor status; Her2 human epidermal growth factor receptor 2; NE not evaluable; Tumor grade, according to Bloom & Richardson scoring method

### FRα expression and concordance between biopsy, primary tumor and metastases in NSCLC patients

**Figure 1:**
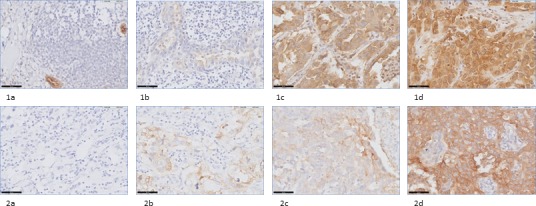
Staining intensities of FRα in NSCLC and breast cancer samples using immunohistochemistry (IHC) The staining score (0 to 3+) was obtained by assessment of membrane intensity. A score of 0 equals no membrane staining, a score of 1+ a faint apical membrane staining, a score of 2+ a moderate apical and occasional lateral membrane staining and a score of 3+ equals a strong circumferential membrane staining. 1a-1d: staining intensity of respectively 0, 1+, 2+ and 3+ in adenocarcinoma samples of NSCLC patients (40x) 2a-d: staining intensity of respectively 0, 1+, 2+ and 3+ in breast cancer samples (40x).

Of all primary tumors containing adenocarcinoma (*N* = 34), 21 out of 34 tumors showed FRα expression of which the majority (>80%) showed overexpression (Figure 1, Table 2). Heterogeneity of FRα expression was seen in 16 out of 21 FRα-expressing adenocarcinomas. Of all primary tumors containing SCC (*N* = 26), only 4 out of 26 tumors showed FRα expression. Overexpression was seen in 1 SCC and a heterogeneous staining pattern in 3 out of 4 SCCs. Of all biopsy specimens (*N* = 23), 8 out of 12 adenocarcinomas showed FRα expression whereas only 2 out of 11 SCCs showed FRα expression. Of all 60 metastatic LNs, obtained from 33 patients, 26 out of 42 LNs containing adenocarcinoma showed FRα expression, whereas 3 out of 18 LNs containing SCC showed FRα expression. Of all distant metastases (*N* = 23), FRα expression was shown in 5 out of 15 adenocarcinomas but in none of the 8 SCCs.

**Table 2:** Folate receptor-α expression in NSCLC

	Total No.	FRα(+) No. (%)	FRα(−) No. (%)	*P*-value
**Biopsies**				
Total	23			
Adenocarcinoma	12	8 (67)	4 (33)	0.036
SCC	11	2 (18)	9 (82)	
**Primary tumor**				
Total	60			
Adenocarcinoma	34	21 (62)	13 (38)	<0.001
SCC	26	4 (15)	22 (85)	
**Lymph node metastases**				
Total	60 (in 33 pts)			
Adenocarcinoma	42 (in 18 pts)	26 (62)	16 (38)	0.004
SCC	18 (in 15 pts)	3 (17)	15 (83)	
**Distant metastasis**				
Total	23			
Adenocarcinoma	15	5 (33)	10 (67)	0.122
SCC	8	0 (0)	8 (100)	

Abbreviations: SCC squamous cell carcinoma; FRα Folate receptor α; pts patients

**Figure 2:**
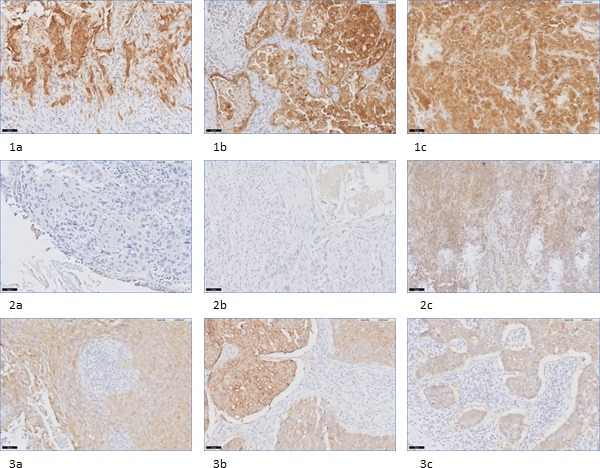
Examples of (dis)concordance in FRα staining in biopsy-, primary tumor-, and metastatic LN tissue in NSCLC and breast cancer patients **1a-1c**: example of concordance between positive FRα expression on biopsy (**1a**), primary tumor (**1b**) and metastatic LN tissue (**1c**) in a NSCLC patient, containing adenocarcinoma (20x). 2a-2c: example of disconcordance between FRα expression on biopsy (**2a**), primary tumor (**2b**) and metastatic LN tissue (**2c**) in a NSCLC patient, containing SCC (20x). **3a-3c**: example of concordance between positive FRα expression on biopsy (**3a**), primary tumor (**3b**) and metastatic LN tissue (**3c**) in a breast cancer patient (20x).

**Figure 3:**
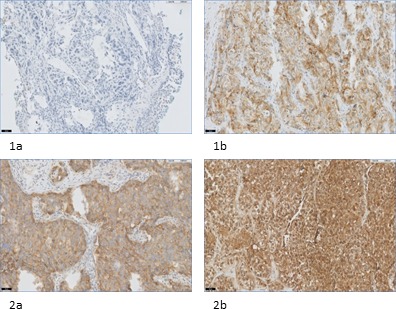
FRα expression in NSCLC and corresponding distant metastases **1a**: primary tumor containing adenocarcinoma without FRα expression (20x). **1b**: corresponding distant metastasis of the bone with positive FRα expression (20x). **2a**: primary tumor containing adenocarcinoma with positive FRα expression (20x). **2b**: corresponding distant metastasis of the brain with positive FRα expression (20x).

Concordance of FRα expression between biopsy and primary tumor was shown in 20 out of 23 biopsies (Figure 2, Table 3). Two of the disconcordances (one adenocarcinoma and one SCC) were attributed to loss of FRα expression in biopsy specimens, while primary tumors did show expression. The other disconcordance was due to upregulation of FRα expression on the biopsy specimen, containing adenocarcinoma. In conclusion, only one biopsy specimen showed false positivity. Concordance between local metastasis, e.g. metastatic LNs, and primary tumor was shown in 31 out of 42 LNs containing adenocarcinoma, within 14 of 18 patients. All disconcordances could be attributed to loss of FRα expression in metastatic LNs. In SCC, concordance of FRα expression between metastatic LNs and primary tumor was achieved in 13 out of 18 LNs, within 12 of 15 patients. In 3 LNs disconcordance was attributed to upregulation of FRα expression, while 2 LNs showed downregulation compared to FRα expression in the primary tumor. Concordance between primary tumors and corresponding distant metastases was seen in 12 out of 15 adenocarcinomas and in 7 out of 8 SCC (Figure 3). Disconcordance in 2 of the metastatic adenocarcinomas and in the metastasis that contained SCC was attributed to downregulation of the distant metastases compared to the primary tumor. The other bone metastasis containing adenocarcinoma showed upregulation compared to the corresponding primary tumor.

**Table 3:** Concordance between biopsy, primary tumor and corresponding disseminated lymph nodes and distant metastases in patients with breast cancer and NSCLC

Degree of concordance	Breast cancer patients No. (%)	NSCLC patients, adenocarcinoma No. (%)	NSCLC patients, SCC, No. (%)
Concordance of FRα status			
Biopsy = Primary tumor	27 out of 40 (67.5)	10 out of 12 (83)	10 out of 11 (91)
Primary tumor = Local metastasis	12 out of 20 (60)	31 out of 42 (78)*	13 out of 18 (80)*
Primary tumor = Distant metastases		12 out of 15 (80)	7 out of 8 (88)
Disconcordance of FRα status			
Biopsy > Primary tumor	2	1	0
Biopsy < Primary tumor	11	1	1
Primary tumor > Local metastasis	6	4	2
Primary tumor < Local metastasis	2	0	3
Primary tumor > Distant metastasis		2	1
Primary tumor < Distant metastasis		1	0

Abbreviations: SCC squamous cell carcinoma; FRα Folate receptor α; pos LN positive lymph node * LN expression was assessed in 33 patients, in respectively 18 patients with adenocarcinoma and 15 patients with SCC; concordance was seen in 14 out of 18 adenocarcinoma patients (31/42 LNs) and 12 out of 15 SCC patients (13/18 LNs).

### FRα expression and concordance between biopsy, primary tumor and metastases in breast cancer patients

Of the total cohort of breast cancer patients in this study (*N* = 40), a positive FRα expression was seen in 12 of the 40 biopsy specimens, 20 of the 40 lumpectomy specimens and 6 of the 20 metastatic LNs (Figure 1, Table 4). Overexpression of FRα was seen in the majority of biopsies, lumpectomy specimens and metastatic LNs, however, almost no homogenous staining patterns were detected. In total, only 5 of the 20 primary tumors showed a homogenous staining pattern. Of all tissue, the hormone receptor (HR) status was known and correlated with FRα expression. As described in Table 4, the HR status, e.g. ER/PR status, showed to correlate negatively with FRα expression of biopsies (*P* = 0.002) and lumpectomy specimens (*P* = 0.010). Of all 15 TN breast cancers that were included in this study, e.g. ER-PR-Her2-, respectively 7 biopsies (*P* = 0.091) and 12 primary tumor specimens (*P* = 0.008) showed a positive FRα expression. In addition, 3 out of 7 metastatic LNs of TN breast cancers showed FRα positivity (*P* = 0.613). Of all biopsy and lumpectomy tissue, a subdivision of FRα expression per LN status was made. Of all specimens of patients with LN metastases (*N* = 27), 7 out of 27 biopsies showed a positive FRα expression and 12 out of 27 lumpectomy specimens.

Concordance of FRα expression between biopsy and primary tumor was shown in 27 out of 40 specimens (Figure 2, Table 3). Disconcordance was attributed to downregulation of FRα on the biopsy specimens compared to the primary tumor in 11 of the 13 tissue sections and upregulation in 2 tissue sections. In conclusion, based on concordance of biopsy and primary tumor tissue, only 2 of the 40 evaluated tissue specimens showed false positivity. Furthermore, in 50% of all tissues, a similar FRα expression was shown in biopsy, lumpectomy and metastatic LN. Concordance between primary tumor and metastatic LN, in the cohort with LN metastases, was seen in 12 out of 20 specimens. Subanalysis showed that 6 out of these 8 disconcordances were attributed to downregulation of FRα expression on metastatic LNs compared to the primary tumor. The remaining two LNs showed a positive FRα expression, whereas the corresponding lumpectomy specimen did not show FRα expression. Separate analysis of the 6 patients that received neoadjuvant therapy showed in 4 out of these 6 patients concordant FRα expression between primary tumor and biopsy specimen. All these 4 patients showed no FRα expression in biopsy nor in primary tumor. The other two patients both showed a positive FRα expression on the primary tumor, but no expression on corresponding biopsy. In 5 of the 6 patients who received neoadjuvant therapy, metastatic LNs were available for evaluation of FRα expression. Only one metastatic LN showed FRα positivity, which was not concordant with the absent FRα expression of the primary tumor.

**Table 4:** Folate receptor-α expression in breast cancer patients

Biopsy	Biomarker status	FRα(+) No. (%)	FRα(−) No. (%)	*P*-value
Total		12	28	
HR status	positive	1 (8)	18 (64)	0.002
	negative	11 (92)	10 (36)	
HER2 status	positive	5 (42)	12 (43)	1.000
	negative	7 (58)	16 (57)	
TN	yes	7 (58)	8 (29)	0.091
	no	5 (42)	20 (72)	
Node status	positive	7 (58)	20 (71)	0.476
	negative	5 (42)	8 (29)	
**Lumpectomy specimen**				
Total		20	20	
HR status	positive	5 (25)	6 (30)	0.010
	negative	15 (75)	14 (70)	
HER2 status	positive	7 (35)	10 (50)	0.523
	negative	13 (65)	10 (50)	
TN	yes	12 (60)	3 (15)	0.008
	no	8 (40)	17 (85)	
Node status	positive	12 (60)	15 (75)	0.501
	negative	8 (40)	5 (25)	
**Lymph node metastasis**				
Total		6	14	
HR status	positive	2 (33)	7 (50)	0.642
	negative	4 (67)	7 (50)	
HER2 status	positive	2 (33)	8 (57)	0.628
	negative	4 (67)	6 (43)	
TN	yes	3 (50)	4 (29)	0.613
	no	3 (50)	10 (71)	

Abbreviations: FRα Folate receptor α; HR hormone receptor including estrogen- and progesterone receptor; HER2 human epidermal growth factor receptor 2; TN triple negative

## DISCUSSION

Since FRα has emerged as target for imaging and treatment purposes, several clinical trials have been conducted with promising results [10, 17]. Diagnostic and therapeutic agents can be delivered intracellular through FRα-mediated endocytosis [18]. During this process, an endosome (containing a FRα-targeted agent bound to FRα) is formed which moves to the recycling center, close to the nucleus. Here, the folate-agent detaches from its receptor and is released, in contrast to the receptor that is brought back to the cell surface. This pathway is mainly responsible for accumulation of FRα-targeted agents in malignant cells. However, absorption of folate in normal tissue such as duodenum and jejunum is facilitated by the proton-coupled folate transporter and the reduced folate carrier (RFC). The latter anion exchanger is responsible for transport of the majority of folate in healthy tissues, while folate-coupled agents show no affinity for this RFC nor the proton-coupled folate transporter [7, 19].

**Figure 4:**
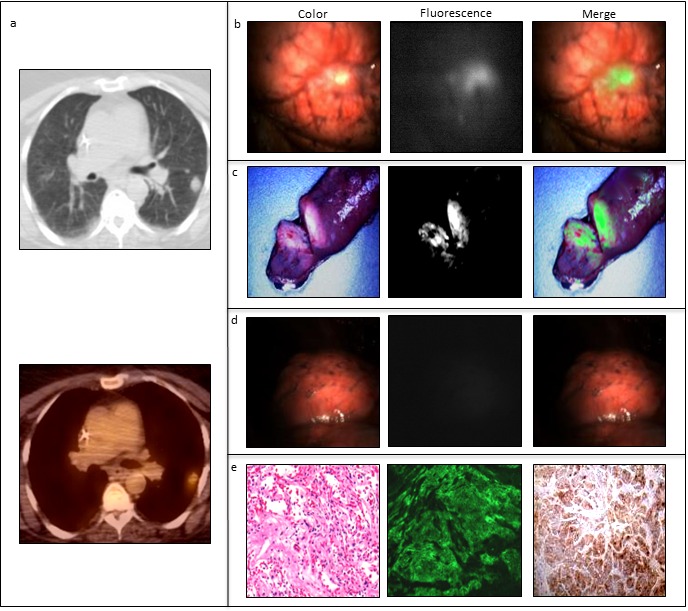
Example of FRα-targeted detection of an adenocarcinoma using EC-17, i.e fluorescent FRα-targeted molecular agent, in a patient suffering from NSCLC. *In vivo* fluorescence imaging was performed using the Artemis imaging system [34]. **a**: Prior to pulmonary resection, a tumor of 3 cm in the upper lobe of the left lung is detected by CT-and PET-scan. **b**: *In vivo* fluorescence imaging shows clear tumor delineation. **c**: *Ex vivo* fluorescence imaging of the tumor in the resected specimen. **d**: After resection, the wound bed was inspected with λex 490 nm and demonstrated no residual fluorescence at the surgical margins. **e**: FRα upregulation was confirmed by fluorescence microscopy and immunohistochemical staining.

As reported by Hilgenbrink and Low et al. [20], folate has been linked to a wide variation of substances used in cancer therapy, including protein toxins, enzymes to activate prodrugs, chemotherapeutics, immunotherapeutic agents, drug-comprising liposomes and nanoparticles. Currently, farletuzumab, vintafolide and IMGN853 are the three FRα-targeting agents that show the most potential in clinical cancer trials, among other as treatment strategy for lung cancer [10, 21]. Detection of malignancies by FRα-targeted imaging approaches is studied broadly using folate linked to radionuclides, PET agents, MRI contrast agents and fluorescent dyes. In NSCLC, a recent study of Okusanya et al. showed feasibility of intraoperative detection of small subpleural lung tumors using a fluorescent FRα-targeted molecular agent, e.g. folate-FITC (Figure 4) [22]. An excellent sensitivity and specificity was shown, resulting in the start of a novel study with the same targeting ligand but optimized fluorophore, e.g. OTL-38 (clinicaltrial.gov). Analysis of a similar clinical trial in breast cancer patients is currently ongoing to determine tumor detection during breast cancer surgery using folate-FITC (clinicaltrial.gov).

In order to efficiently apply both FRα-targeted therapeutic and imaging agents, adequate patient selection regarding FRα expression is required. In addition, knowledge about the concordance of FRα expression between biopsy, primary tumor specimen and (possible) metastasis is pivotal to estimate efficacy and usability of these approaches. Based on results from the current study, selection of breast cancer and NSCLC patients who might benefit from FRα-targeted approaches can be performed reliably *via* biopsy staining. Although the majority of tumor specimens showed heterogeneity of FRα expression, less than 5% of all biopsies showed false positivity. In a clinical setting, this suggests that 1 out of 20 included breast cancer or NSCLC patients in a FRα-targeted trial would show no FRα expression in the primary tumor while the biopsy stained FRα-positive. Regarding the targeting of disseminated malignant cells, this study shows high concordance rates of local/distant metastases with corresponding primary tumors. Although concordance rates were relatively lower for breast cancer than for NSCLC, e.g. 68% *vs*. >80%, these results still suggest that the majority of metastases can be targeted when the primary tumor shows FRα expression.

Overall, disconcordance was mainly due to downregulation of FRα in biopsy- and metastastic tissue compared to primary NSCLC and breast cancer, which could be a result of tissue sampling or of heterogeneity of FRα expression. In this study, whole tissue slides were used to overcome the difficulty in evaluating heterogeneity as is experienced with the generally used TMAs [23]. Of all 266 evaluated tumor tissue specimens, heterogeneity of FRα was shown in the majority (>80%) of the stained sections.

Several IHC studies investigated FRα expression on primary lung (NSCLC) and breast cancers. Two lung studies applied the same monoclonal antibody as used in this study, i.e. 26B3.F2, and displayed similar expression rates, underscoring the validity of the used staining: more than 70% of adenocarcinomas expressed FRα and less than 15% of SCCs [14, 24]. In the current study, a significant association between FRα expression and histological subtype was shown in biopsies (*P* = 0.036), primary tumors (*P* < 0.001) and metastatic LNs (*P* = 0.004). The distinction in FRa expression between adenocarcinoma and SCC is caused by the type of cancer cell they derive from; adenocarcinoma originates from FRα-expressing type 1 and 2 pneumocytes and SCC from more centrally positioned tracheal cells which do not express the receptor [14, 25].

Other IHC studies using a different monoclonal antibody (mAB343) showed expression rates in adenocarcinoma and SCC ranging from 72% to 87% and 15% to 57% respectively [13, 26]. The difference in percentages of positive FRα expressing tumors between studies may be partially contributed to a variation in applied antibodies. For example, relatively high percentages of FRα expression in patients with SCC obtained using mAB343 may be a result of non-specific binding [13, 26]. Furthermore, cut-off values for defining FRα positivity and scoring methods are diverse amongst studies. Nunez et al. [13] examined FRα expression in NSCLC patients (*n* = 320) by using the H-score, which offers a separate score for both cytoplasmic and membranous staining. In breast cancer, cut-off values for defining FRα positivity differed from 5-15%. Scoring using the M-score has been described in which both membranous tumor cell staining and the proportion and staining intensity of FRα positive cells are captured [16]. Hartmann et al. [27] assessed FRα expression using mAB343 on a tissue micro array (TMA) containing samples of 63 invasive breast cancers with either poor or good outcome. An association between strong FRα expression and a poor outcome, defined as median time to recurrence of 1.9 years, was shown. The latter results are in line with other studies describing a significant association between FRα expression and worse (disease-free) survival [16, 28]. Increased folate storage in FRα-expressing tumors may cause accelerated growth and consequently, a more aggressive tumor. However, a recent study describing FRα expression in breast cancer brain metastases was unable to show any association between FRα expression and survival, possibly due to small sample size [29]. In lung cancer, results from studies investigating survival and FRα expression also vary. Several IHC studies and a study investigating FRα gene expression using RT-PCR reported an improved overall survival when FRα expression is high [14, 30]. However, Nunez et al. could not show any correlation between FRα expression and overall- or recurrence free survival in a cohort of 320 patients [13]. It still remains unclear why high levels of FRα are correlated with a favorable prognosis; further research is warranted to explain this.

Recent studies showed that FRα expressing breast cancers represented a novel molecular subtype associated with ER-/PR-/Her2Neu- and ER- breast cancers [16, 28]. The current study endorses these conclusions as 12 out of 15 primary tumors of TN breast cancers showed a positive FRα expression and a significant correlation between HR status and FRα expression on both biopsy specimens (*P* = 0.002) and primary tumors (*P* = 0.010) was found. It is known that estrogens are involved in the regulation of FRα expression and 17β estradiol *via* the P4 promoter, resulting in down-regulation of FRα [31, 32]. Positive FRα expression was shown in respectively 30%, 50% and 30% of biopsy-, lumpectomy- and metastatic LN tissue. Although this percentage is higher than described in literature, it must be noted that 67.5% of the included patients had LN metastases (N1-2) and 37.5% were TN breast cancer patients. Furthermore, tumors displayed a relatively high tumor grade. In the study of Zhang et al. [28], a significant association between FRα positive expression and high histologic grade, high nodal stage and subgroups of ER/PR- and TN breast cancers was shown. In an extra cohort of metastatic stage IV, Her2 negative breast cancer patients, O'shannessy et al. [16] described that the percentage of FRα positive tumors in early stage disease is retained in late stage metastatic disease, as 36% of samples showed FRα positive expression of whom 86% were TN breast cancers.

In an additional cohort of NSCLC patients (*N* = 23), concordance of FRα expression between primary tumor and corresponding distant metastases was assessed. In the literature, little is known about FRα expression on distant metastases. In a study of O'shannessy et al. [14], 9 fine needle aspirates comprising LN metastases of lung adenocarcinoma showed 63% FRα positivity. Furthermore, Nunez et al. [13] reported no significant difference in FRα expression between 27 NSCLC and 15 derived metastatic sites. We show concordance rates between primary tumor and distant metastases of respectively 80% and 88% for adenocarcinomas and SCCs. In addition, all metastatic LNs showed concordant FRα expression with the corresponding distant metastases. On the contrary, Kikuchi et al. [33] showed downregulation of FRα gene expression in 16 metastatic brain tumors compared to 22 primary lung adenocarcinomas using cDNA microarray. However, as this study investigated gene expression and non-corresponding metastases, results have to be interpreted with caution.

**Figure 5:**
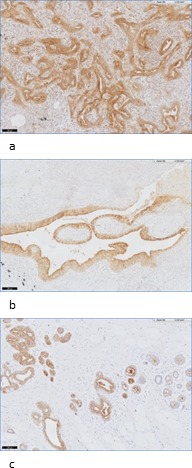
Examples of FRα staining in normal lung and breast tissue **a**: staining of FRα at the luminal border of normal lung tissue in an adenocarcinoma patient (10x). **b**: staining of FRα at the luminal border of normal lung tissue in a SCC patient (10x). **c**: staining of FRα in normal breast tissue: staining at the luminal border of secretory cells (10x).

Finally, normal breast and lung cells showed FRα expression (Figure 5). In particular the luminal border of secretory cells (due to the secretion of folate in milk) and the myoepithelial layers of normal epithelial breast tissue stained positive. Nonetheless, FRα expression on normal breast and lung tissue will not significantly interfere when FRα-targeted agents are intravenously administered, since the receptor is confined to the luminal side of polarized epithelial cells. The lack of cellular polarity on malignant cells on the other hand, will lead to binding of FRα targeted agents through the whole tumor [18].

In conclusion, this study shows high concordance rates of FRα expression between primary tumor and corresponding biopsy and metastatic tissue of both breast cancer and NSCLC patients, underscoring applicability of FRα-targeted agents in these patients. Moreover, an association between FRα expression and histological/molecular subtype of tumors was shown. Importantly, false positivity of FRα expression on biopsies was limited to less than 5%. Although this study is performed with a relatively small amount of tissue specimens, the current results provide rational for the use of biopsies to select lung and breast cancer patients for FRα-targeted agents, eventually leading to optimal personalized medicine.

## MATERIALS AND METHODS

### Tissue samples

The total cohort of NSCLC patients consisted of 38 patients who received curative surgery between 2011-2013 (*N* = 38) and 22 patients with distant metastases (2000-2013). Of all 60 NSCLC patients, 34 primary tumors containing adenocarcinoma and 26 SCCs were included as shown in Table 1. To assess the correlation between FRα expression on primary tumor and preoperatively obtained biopsy tissue, all available biopsy specimens, obtained *via* mediastinal biopsy, were collected. However, in some patients a transesophageal endoscopic ultrasound fine needle aspiration (EUS-FNA) or endobronchial ultrasound transbronchial needle aspiration (EBUS-TBNA) was performed and some patients were referred to our academic center, of whom biopsy specimen could not be obtained. In total, 23 biopsies were collected: 12 biopsies containing adenocarcinoma and 11 biopsies containing SCC (Table 2). Moreover, 60 metastatic LNs of 33 patients were stained for FRα. Adenocarcinoma was present in 42 of these LNs, while SCC was present in the remaining 18 metastatic LNs. Fifteen of the distant metastases contained adenocarcinoma, whereas 8 of them contained SCC. Of one patient who suffered from adenocarcinoma, tissues from two different metastatic sites were included. Metastases originated mostly from the brain (*n* = 10), followed by metastases from bone (*n* = 7), liver (*n* = 3), skin (*n* = 1), adrenal gland (*n* = 1) and jejunum (*n* = 1).

The total cohort of breast cancer patients consisted of 40 patients, who had undergone a breast conserving surgery (lumpectomy) for a cT1 or cT2 stage tumor between 2000-2014. In total, 6 of the 40 patients received neoadjuvant chemotherapy, of whom obtained results are separately described. The first cohort of breast cancer patients consisted of 20 patients of whom biopsy specimen and lumpectomy tissue was stained. In addition, a second cohort of 20 patients was included to assess concordance of FRα expression between biopsy specimen, lumpectomy and metastatic LNs. Of all patients, the receptor status of the tumor tissue was known, e.g. ER, PR and HER2 status.

### Immunohistochemistry

IHC was performed using formalin-fixed, paraffin-embedded (FFPE) tissue, obtained from the Pathology archive from the Leiden University Medical Center, Diakonessen Hospital and Rijnland Hospital. All samples were handled in an anonymous fashion according to the national ethical guidelines (‘Code for Proper Secondary Use of Human Tissue,’ Dutch Federation of Medical Scientific Societies) and approved by the Institutional Ethics Committee of the Leiden University Medical Center. Tissue samples were stained using the monoclonal antibody (Mab) 26B3.F2 (certified Folate Receptor alpha IHC Assay Kit, Biocare Medical) and subsequently scored for staining. The Mab26B3.F2 is highly specific for FRα without cross-reactivity to the other FR, e.g. FRβ, FRγ or FRδ. Validation of the staining protocol was performed by using (the recommended) lung adenocarcinoma as positive control and normal liver as negative control. Immunohistochemical staining was performed on 4 um paraffin sections on adhesive slides. Slides were deparaffinized in xylene and rehydrated in decreasing concentrations of ethanol. After rinsing in distilled water, endogenous peroxidase was blocked with hydrogen peroxide for five minutes. Slides were rinsed in water and antigen retrieval was performed in the DAKO PT Link, Target Retrieval Solution pH6.0 at 95°C for 10 minutes. After rinsing in PBS, nonspecific sites were blocked with a protein block (Background Punisher) for 5 minutes and the secondary antibody was incubated for 30 minutes on room temperature. As a negative control, one control slide was incubated with negative control reagent. After three washes the slides were incubated with MACH4 Mouse Probe Primary Antibody Enhancer (Biocare Medical) for 10 minutes. Again slides were washed in PBS, followed by incubation with MACH4 HRP Polymer for 10 minutes. Subsequently, the slides were washed and antibody binding was visualized by using 3,3′-diaminobenzidine (DAB, DAKO). Sections were counterstained with haematoxylin, dehydrated and mounted with pertex.

### Scoring method

FRα expression was assessed in both lung and breast cancer tissue by using a membranous scoring method with a scale ranging from 0 to 3+, as described by O'shannessy et al. [14]. A score of 0 corresponded with absence of staining; 1+ equaled faint staining on luminal borders; 2+ equaled moderate staining on apical and sometimes lateral borders and 3+ indicated strong circumferential staining (Figure 1). The tumor was considered positive when more than 10% of malignant cells were positively stained (>0). Overexpression of FRα was defined as a score of 2+ or 3+. Homogeneity was described when FRα expression was detectable on all malignant cells. Concordance was achieved when the expression score of one of the stained tissues, i.e. biopsy or corresponding LN or distant metastasis, matched the score of the primary tumor and both displayed either positive or negative expression of FRα. In general, FRα expression of the primary tumor specimen was considered as golden standard. Concordance between either biopsy or metastatic tissue with the primary tumor was described as down- or upregulation of FRα. False positive expression was determined as positive FRα expression on biopsy or metastatic tissue without FRα expression on the primary tumor. Evaluation of the immunohistochemical staining was performed blinded independently by two observers. Disagreements were resolved by consensus after reviewing the relevant slide with the pathologist.

### Statistical analysis

The statistical analysis was performed using SPSS version 22.0 software (SPSS, ©IBM Corporation, Somer NY, USA). FRα expression per histopathological subgroup was calculated by the Fishers exact test. A P-value of < 0.05 was considered statistically significant.
